# A strain rate dependent thermo-elasto-plastic constitutive model for crystalline metallic materials

**DOI:** 10.1038/s41598-021-88333-1

**Published:** 2021-04-23

**Authors:** Cen Chen, TzuChiang Wang

**Affiliations:** 1 State Key Laboratory of Nonlinear Mechanics, Institute of Mechanics, Chinese Academy of Sciences, Beijing, 100190 China; 2 School of Engineering Sciences, University of Chinese Academy of Sciences, Beijing, 100049 China

**Keywords:** Metals and alloys, Theory and computation

## Abstract

The strain rate and temperature effects on the deformation behavior of crystalline metal materials have always been a research hotspot. In this paper, a strain rate dependent thermo-elasto-plastic constitutive model was established to investigate the deformation behavior of crystalline metal materials. Firstly, the deformation gradient was re-decomposed into three parts: thermal part, elastic part and plastic part. Then, the thermal strain was introduced into the total strain and the thermo-elastic constitutive equation was established. For the plastic behavior, a new relation between stress and plastic strain was proposed to describe the strain rate and temperature effects on the flow stress and work-hardening. The stress–strain curves were calculated over wide ranges of strain rates (10^–6^–6000 s^−1^) and temperatures (233–730 K) for three kinds of crystalline metal materials with different crystal structure: oxygen free high conductivity copper for face centered cubic metals, Tantalum for body centered cubic metals and Ti–6Al–4V alloy for two phase crystal metals. The comparisons between the calculation and experimental results reveal that the present model describes the deformation behavior of crystalline metal materials well. Also, it is concise and efficient for the practical application.

## Introduction

The strain rate and temperature effects on the deformation behavior of crystalline metal materials have been investigated deeply for many years^1–3^. The experimental results show that the yield stress increases with the strain rate rising and decreases with the temperature rising for crystalline metals^4–11^. While, the working-hardening reduces with the increase of strain rate and temperature for many crystalline metals, such as Ti–6Al–4V and tantalum^12,13^. It is also found that the plastic behaviors of crystalline metals display a non-linearly dependent on the strain rate and temperature. To describe the deformation behavior of crystalline metals at different strain rates and temperatures, many constitutive models had been established in the past years. Simple uniaxial stress–strain model and one-dimensional stress wave propagation model had been proposed initially to describe the temperature and strain rate effects on the flow stress^14,15^. Then, some phenomenological models had been developed sequentially. Amongst these theoretical research, Johnson–Cook (JC)^2^, Zerilli–Armstrong (ZA)^16,17^ and Khan–Huang–Liang (KHL)^18^ models have been well accepted due to their predictive ability for the viscoplastic behavior of crystalline metals under wide ranges of strain rate and temperature. The JC model was introduced to build a constitutive equation for the strain rate sensitivity and softening effect on the work-hardening behavior at different temperatures for crystalline metals^2^. The ZA model used the dislocation mechanics concept for different face centered cubic (fcc) and body centered cubic (bcc) materials over different strain rates between room temperature and 0.6 *T*_*m*_ (*T*_*m*_: melt temperature). The KHL model was developed to describe the viscoplastic hardening behavior of polycrystalline materials and had successfully described the work-hardening behavior of copper (Cu) under cyclic shear straining and biaxial tension–torsion^18^. Although these models mentioned above describe the strain rate and temperature effects on the flow softening and strain hardening behaviors, there are still some limitations for them. For example, the strain rate and temperature effects on the flow stress are uncoupled in the JC model which is not in line with the case of most crystalline metals; the ZA model is not effective to predict the plastic behavior at temperatures above 0.6 *T*_*m*_ and lower strain rate, and it considers two different forms for fcc and bcc materials.

Also, the experimental results showed that the temperature effect on the elastic behavior should not be ignored due to the free thermal expansion of lattice and the thermal vibration of atoms^19^. Since that the thermal vibration of atoms can only be revealed by the form of heat energy and the structural deformation is explicitly modeled at the continuum level, the thermal vibration was not considered simultaneously with the structural deformation in the phenomenological models. Therefore, the focus of these phenomenological models was on the description of work-hardening and flow stress at different strains, strain rates, and temperatures. Although the atomistic models can describe the thermal vibration and the structural deformation can be reflected by the total atomic motion^20^, the normal vibration frequency should be recomputed when the elastic strain changes, which results in a low calculation efficiency^21^.

Hence, a desirable constitutive model should be widely applicable and capable to describe the thermo-elasto-plastic deformation behavior under different temperatures and strain rates. Then, the flexibility of determining calculated parameters from a limited set of experimental data and the accuracy of the calculation results for different material under static and/or dynamic are also important factors in judging the success of a new model. In this paper, a strain rate dependent thermo-elasto-plastic constitutive model was proposed based on a new decomposition of deformation gradient. The stress–strain curves were calculated for Oxygen Free High Conductivity (OFHC) fcc Cu, bcc Tantalum (Ta) and two phase Ti–6Al–4V alloy from 233 to 730 K at different strain rates. The comparison between the calculation and experimental results shows that the present model describes the thermo-elasto-plastic deformation at different strain rates and temperatures accurately. And it is easily to applied in different crystalline metal materials.

## Methods

### New decomposition of deformation gradient

**Figure 1:**
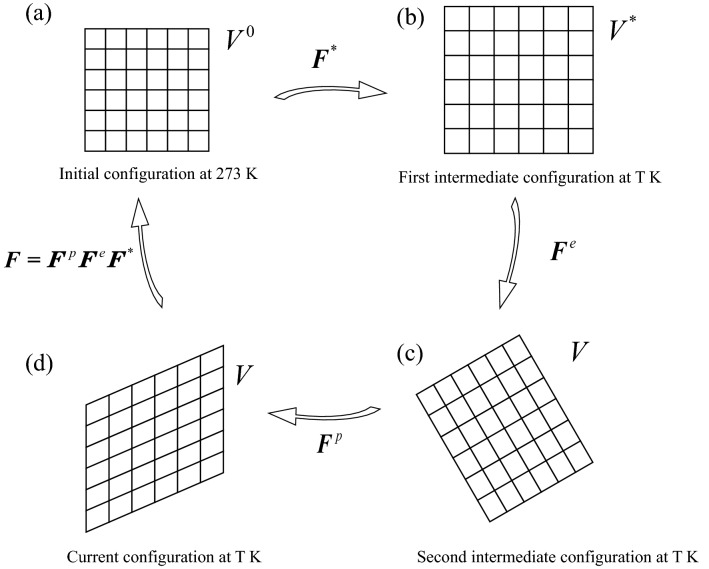
Decomposition of deformation configuration: (**a**) initial configuration; (**b**) first intermediate configuration; (**c**) second intermediate configuration; (**d**) current configuration.

Different from the kinematical theory, which decomposed the deformation gradients into plastic and elastic parts^22–24^, the deformation gradient ***F*** in the present model was re-decomposed in to thermal, elastic and plastic parts, which is written as

1 $${\varvec{F}} = F^{p} F^{e} F^{*} ,$$

where ${\varvec{F}}^{p}$ is the plastic deformation gradient, ${\varvec{F}}^{e}$ is the elastic deformation gradient, and ${\varvec{F}}^{*}$ is the thermal deformation gradient caused by the free thermal expansion. The whole deformation process is described in four configurations. Firstly, the undeformed state at 273 K is defined as the initial configuration (Fig. 1a). Secondly, the first intermediate configuration is defined at the state after free thermal expansions at T K (Fig. 1b). Then, the second intermediate configuration is defined at the state after elastic deformation at T K (Fig. 1c). Lastly, the current configuration is the state after plastic deformation at T K (Fig. 1d).

### Decomposition of total strain

Based on the new decomposition of deformation gradient, the total strain tensor is expressed as

2 $$\begin{aligned} {\varvec{E}} & = \frac{1}{2}\left( {{\varvec{F}}^{{\text{T}}} {\varvec{F}} - I} \right) \\ & = \frac{1}{2}\left[ {\left( {{\varvec{F}}^{*T} {\varvec{F}}^{{e{\text{T}}}} {\varvec{F}}^{{p{\text{T}}}} {\varvec{F}}^{p} {\varvec{F}}^{e} {\varvec{F}}^{*} } \right) - {\varvec{I}}} \right] \\ & = {\varvec{E}}^{*} + {\varvec{F}}^{{*{\text{T}}}} {\varvec{E}}^{e} {\varvec{F}}^{*} + {\varvec{F}}^{{*{\text{T}}}} {\varvec{F}}^{{e{\text{T}}}} {\varvec{E}}^{p} {\varvec{F}}^{e} {\varvec{F}}^{*} . \\ \end{aligned}$$

The deformation gradients ${\varvec{F}}^{*}$ and ${\varvec{F}}^{e}$ can be written as ${\varvec{F}}^{*} = {\varvec{R}}^{*} {\varvec{U}}^{*}$ and ${\varvec{F}}^{e} = {\varvec{R}}^{{\text{e}}} {\varvec{U}}^{{\text{e}}}$, where ${\varvec{R}}^{*}$ and ${\varvec{R}}^{e}$ are the rotation tensors, ${\varvec{U}}^{*}$ and ${\varvec{U}}^{{\text{e}}}$ are the symmetrical stretch tensors. Assuming that ${\varvec{R}}^{*} = {\varvec{I}}$, ${\varvec{R}}^{e} = {\varvec{I}}$, the total strain tensor takes form as

3 $${\varvec{E}} = {\varvec{E}}^{*} + {\varvec{U}}^{*} {\varvec{E}}^{e} {\varvec{U}}^{*} + {\varvec{U}}^{*} {\varvec{U}}^{e} {\varvec{E}}^{p} {\varvec{U}}^{e} {\varvec{U}}^{*} .$$

Considering that the thermal strain tensor ${\varvec{E}}^{*}$ and the elastic strain tensor ${\varvec{E}}^{e}$ are usually small enough during the deformation process, the total strain tensor is written as^25^

4 $${\varvec{E}} = {\varvec{E}}^{*} + {\varvec{E}}^{e} + {\varvec{E}}^{p} .$$

Equation (4) is a new expression for the total strain tensor consisting of thermal, elastic and plastic parts, and more details about the new decomposition of deformation Gradient and total strain were displayed in our early work^25^. Although the deformation gradient can be decomposed as ${\varvec{F}} = {\varvec{F}}^{ * } {\varvec{F}}^{e} {\varvec{F}}^{p}$ or ${\varvec{F}} = {\varvec{F}}^{e} {\varvec{F}}^{p} {\varvec{F}}^{ * }$, the decomposition ${\varvec{F}} = {\varvec{F}}^{p} {\varvec{F}}^{e} {\varvec{F}}^{ * }$ is better than the others since it can get a brief mathematical expression for total strain as Eq. (4) and it is more aligned with the actual deformation process.

### Thermal strain

The thermal strain tensor ${\varvec{E}}^{*}$ for the crystalline metal materials is expressed as

5 $${\mathbf{E}}^{*} = \left[ {\begin{array}{*{20}c} {\varepsilon_{T} } & 0 & 0 \\ 0 & {\varepsilon_{T} } & 0 \\ 0 & 0 & {\varepsilon_{T} } \\ \end{array} } \right].$$

The thermal strain $\varepsilon_{T}$ at temperature *T* is obtained by^26^

6 $$\varepsilon_{T} = \int\limits_{{T_{0} }}^{T} {\alpha {\text{d}}T} ,$$

where *T*_*0*_ = 273 K, the coefficient of thermal expansion *α* can be obtained easily by the experimental results^27^ or theoretical method^28^.

### Thermo-elastic constitutive equation for single crystals

The second Piola–Kirchhoff stress is written as

7 $${\varvec{S}} = \frac{\partial W}{{\partial {\varvec{E}}^{e} }} = \frac{1}{{V^{*} }}\left[ {\frac{{\partial U_{E} \left( {{\varvec{E}}^{e} } \right)}}{{\partial {\varvec{E}}^{e} }}} \right],$$

where V^*^ is the volume at the first intermediate configuration in Fig. 1b, *U*_*E*_ is the elastic strain energy in the unit volume.

Then, the constitutive equation that expressed by the rate of the second Piola–Kirchhoff stress and the rate of Green strain is

8 $$\dot{\user2{S}} = \frac{1}{{V^{*} }}\left[ {\frac{{\partial U_{E}^{2} \left( {{\varvec{E}}^{e} } \right)}}{{\partial {\varvec{E}}^{e} \partial {\varvec{E}}^{e} }}} \right]:\dot{\user2{E}}^{e} = \frac{1}{{V^{*} }}\left[ {\frac{{\partial U_{E}^{2} \left( {{\varvec{E}}^{e} } \right)}}{{\partial {\varvec{E}}^{e} \partial {\varvec{E}}^{e} }}} \right]:(\dot{\user2{E}} - \dot{\user2{E}}^{p} - \dot{\user2{E}}^{*} ).$$

The thermo-elastic stiffness tensor for single crystals is

9 $${\varvec{C}}^{sig} { = }\frac{1}{{V^{*} }}\left[ {\frac{{\partial U_{E}^{2} \left( {{\varvec{E}}^{e} } \right)}}{{\partial {\varvec{E}}^{e} \partial {\varvec{E}}^{e} }}} \right].$$

Also, the Cauchy stress is written as

10 $${\varvec{\sigma}} = \frac{1}{V}{\varvec{F}}\left[ {\frac{{\partial U_{E} \left( {{\varvec{E}}^{e} } \right)}}{{\partial {\varvec{E}}^{e} }}} \right]{\varvec{F}}^{{\text{T}}} = \frac{{V^{*} }}{V}{\varvec{FSF}}^{{\text{T}}} = \frac{1}{J}{\varvec{FSF}}^{{\text{T}}} ,$$

where *V* is the volume at the second intermediate configuration in Fig. 1c, and $J = \frac{V}{{V^{*} }}$.

### Thermo-elastic stiffness for polycrystalline metal materials

Assuming that the Polycrystalline material is the aggregate of randomly oriented single crystals, and the orientation of single crystal is specified by the Euler angles $(\theta ,\varphi ,\psi )$^25,29^. Then, the thermo-elastic stiffness for the polycrystalline material is calculated by the stiffness of single crystal with various directions, which is expressed as

11 $$C_{ijkl}^{pol} = \int\limits_{0}^{\pi } {\int\limits_{0}^{2\pi } {\int\limits_{0}^{2\pi } {\left( {R_{im} } \right)^{ - 1} \left( {R_{jn} } \right)^{ - 1} \left( {R_{kp} } \right)^{ - 1} \left( {R_{lq} } \right)^{ - 1} C_{mnpq}^{sig} f\left( {\theta ,\varphi ,\psi } \right)\sin \theta } } } \;d\psi \;d\varphi \;d\theta ,$$

where *R*_*i j*_ is the component of rotation tensor*** R ***by the Euler angles^29^, $C_{mnpq}^{sig}$ is the component of thermo-elastic stiffness tensor for the single crystals in Eq. (9), and *f (θ, ϕ, ψ)* is the orientation distribution function for single crystals. More details for calculating the thermo-elastic stiffness of polycrystalline were display in our early work^25^.

### Plastic constitutive equation

In the previous studies^30–33^, the power law is adopted to describe the plastic behavior of metallic materials. In the present work, a new relation between the stress and plastic strain is proposed to reflect the strain rate and temperature effects on the plastic behavior. After exceeding the yield point, the stress at the given temperature and strain rate is expressed as

12 $$\sigma = \left( {B\left( {\frac{{\dot{\varepsilon }}}{{\dot{\varepsilon }_{r} }}} \right)^{n} + \varepsilon_{{^{p} }}^{m} } \right)f(T,\dot{\varepsilon }),$$

where *m* is strain-hardening index (0 ≤ *m* ≤ 1); *B* and *n* are material parameters that relate to yield strength; $\dot{\varepsilon }_{r}$ is reference strain rate. $f(T,\dot{\varepsilon })$ reflects the temperature and strain rate effects on the flow stress and work-hardening, which is expressed as

13 $$f(T,\dot{\varepsilon }) = A\left( {1 + C\left( {\frac{T}{{T_{r} }}} \right)^{\gamma } } \right)\left( {1 + P_{1} \left( {\frac{{\dot{\varepsilon }}}{{\dot{\varepsilon }_{r} }}} \right) + P_{2} \left( {\frac{{\dot{\varepsilon }}}{{\dot{\varepsilon }_{r} }}} \right)^{\beta } } \right),$$

where, *A*, *C*, *P*_1_, *P*_2_, *γ* and *β* are the material parameters. *T*_*r*_ is the reference temperature.

Then, the relation between stress and plastic strain is written as

14 $$\sigma = A\left( {B\left( {\frac{{\dot{\varepsilon }}}{{\dot{\varepsilon }_{r} }}} \right)^{n} + \varepsilon_{{^{p} }}^{m} } \right)\left( {1 + C\left( {\frac{T}{{T_{r} }}} \right)^{\gamma } } \right)\left( {1 + P_{1} \left( {\frac{{\dot{\varepsilon }}}{{\dot{\varepsilon }_{r} }}} \right) + P_{2} \left( {\frac{{\dot{\varepsilon }}}{{\dot{\varepsilon }_{r} }}} \right)^{\beta } } \right).$$

### Determination of parameters

At yield point, *ε*_*p*_ = 0, the yield stress for a given strain rate and temperature can be obtained by Eq. (12) as follow:

15 $$\sigma_{{{\text{ys}}}} = B\left( {\frac{{\dot{\varepsilon }}}{{\dot{\varepsilon }_{r} }}} \right)^{n} f(T,\dot{\varepsilon }).$$

Then, the stress is written as

16 $$\sigma = \sigma_{ys} + \varepsilon_{{^{p} }}^{m} f(T,\dot{\varepsilon }).$$

Equation (16) is reduced to

17 $$\ln (\sigma - \sigma_{ys} ) = m\ln \varepsilon_{p} + \ln (f(T,\dot{\varepsilon })).$$

By the stress–strain curve, the yield stress can be obtained easily. Then, using the plot of $\ln (\sigma - \sigma_{ys} )$ VS $\ln \varepsilon_{p}$, the value of $f(T,\dot{\varepsilon })$ is found from the interception with the vertical axis and *m* is from the slope. Lastly, the other parameters are calculated easily by the mathematical software Matlab when the values of yield stress and $f(T,\dot{\varepsilon })$ are obtained at the given strain rate and temperature.

## Results

Applications of the present model as well as comparisons to the experimental data for OFHC Cu^34,35^, Ta^12,36^ and Ti–6Al–4V alloy^13,37^ metals are illustrated in this section. The OFHC Cu, as an important fcc metal, is widely used in the industry due to its high ductility combined with low volatility, high thermal and electrical conductivity. Ta is a bcc metal that has generated a lot of interest in industry due to its density, strength and ductility over wide ranges of strain rates and temperatures. Ti–6Al–4V alloy, as the most widely used Ti alloy, has been applied in aero-engine, gas turbines and other applications due to their high strength to weight ratio, ductility, and ability to withstand high temperatures and resist corrosion. It consists of hcp α-grains, with a dispersion of stabilized bcc β phase around grain boundaries at room temperature. And α phase transforms to β phase at 873 K^13^. The Cu, Ta and Ti–6Al–4V alloy are used here as the applications for the present model to show the temperature and strain rate effects on the elastic deformation, flow stress and work-hardening for metal with different crystal structure.

We calculated the stress–strain curves of these three kinds of materials at different temperatures and strain rates. For Cu, the stress-plastic strain curves have been calculated from 294 to 730 K with strain rate from 0.0004 to 6000 s^−1^^34,35^. For Ta, the temperature is from 298 to 589 K, and the strain rate is from 10^–6^ to 0.53 s^−1^^12,36^. And for Ti–6Al–4V alloy, the strain rate is from 10^–5^ to 2700 s^−1^, and the temperature is from 233 to 598 K^13,37^. The calculated parameters were displayed in the Table 1. The reference strain rate $\dot{\varepsilon }_{r}$ is 1 s^−1^, and the reference temperature *T*_*r*_ is 298 K.

**Table 1:** Material parameters for the present constitutive model.

	A/MPa	B	m	n	C	P_1_	P_2_	γ	β
OFHC Cu	1456.11	0.09	0.38	0.00	101.92	− 3.75 × 10^–8^	− 1.00	− 0.49	− 5.71 × 10^–5^
Ta	350.41	2.05	0.30	0.21	0.69	0.060	− 0.81	− 3.50	0.058
Ti–6Al–4V alloy	75.23	0.88	0.60	0.062	519.51	− 8.89 × 10^–7^	− 0.97	− 0.60	7.42 × 10^–4^

**Figure 2:**
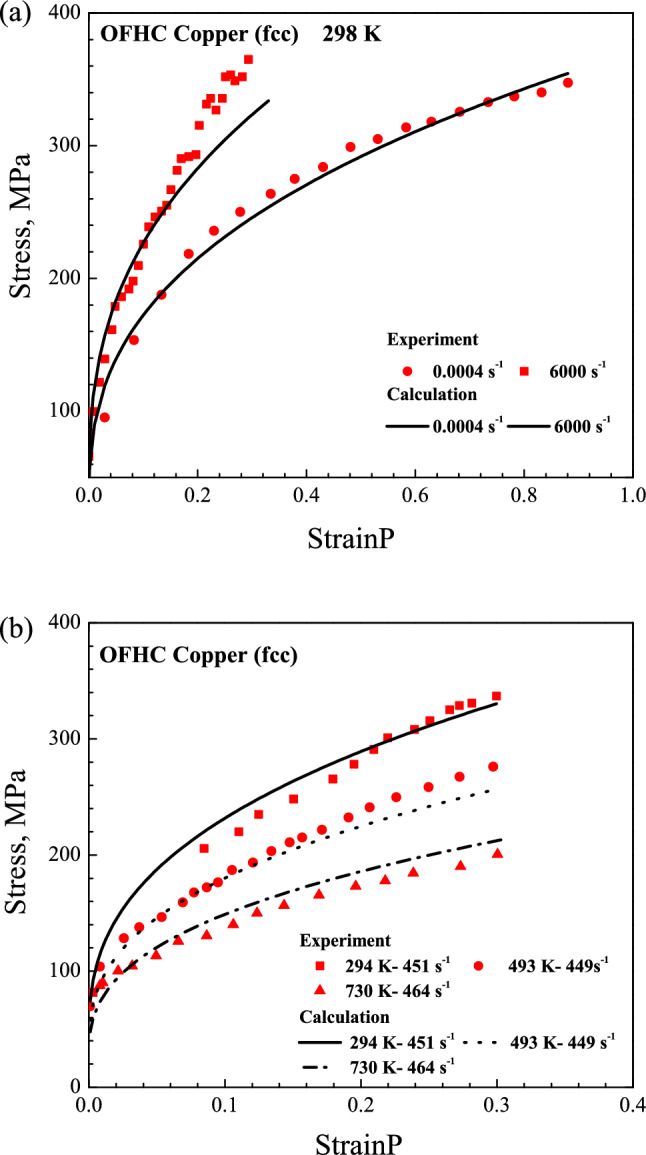
Comparison of stress–plastic strain curves for OFHC Cu between the calculated and experimental results at different temperatures and strain rates.

**Figure 3:**
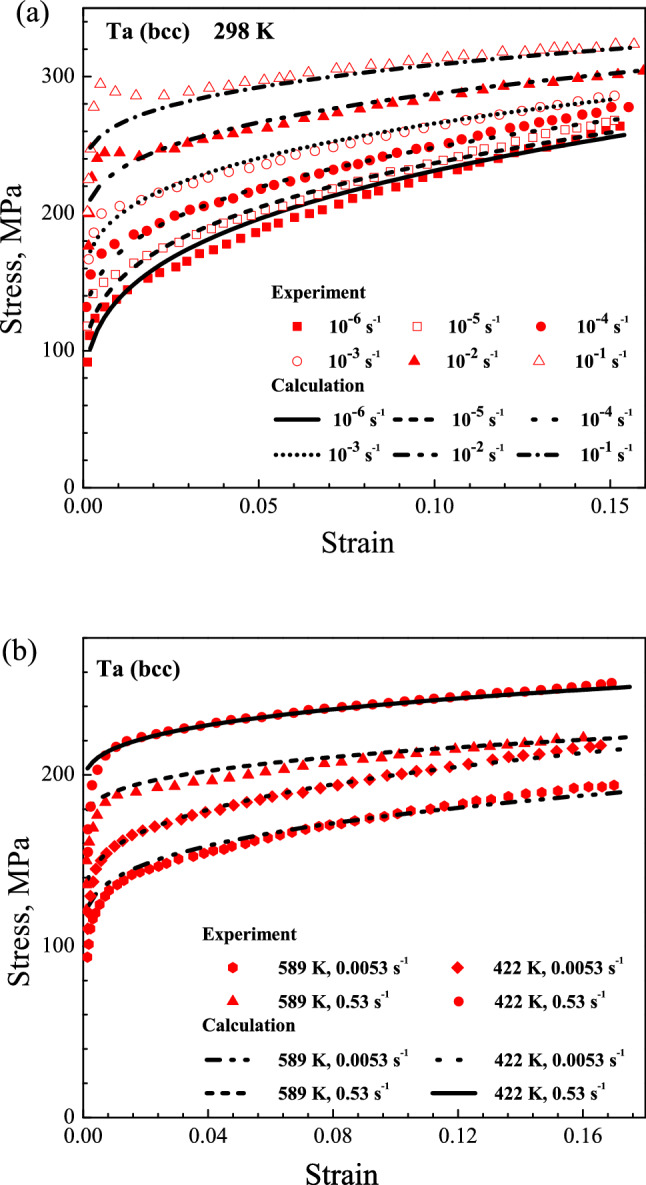
Comparison of stress–strain curves for Ta between the calculated and experimental results at different temperatures and strain rates.

**Figure 4:**
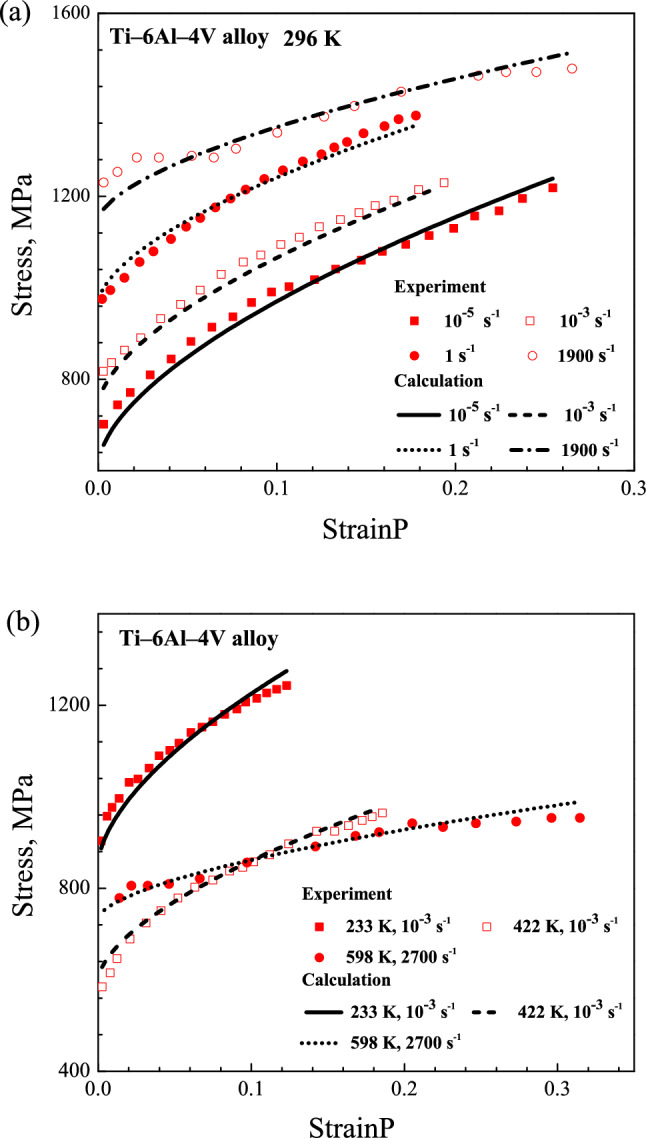
Comparison of stress–plastic strain curves for Ti–6Al–4V alloy between the calculated and experimental results at different temperatures and strain rates.

The calculation and experiment results are displayed in the Figs. 2, 3 and 4. The results display that the yield stress and work-hardening behavior exhibit a high sensitivity to strain rate and temperature for Cu, Ta and Ti–6Al–4V alloy. It is found that the yield stress decreases with temperature rising but increases with strain rate rising. At the same time, the work-hardening decreases with the increasing of temperature and strain rate. For the example of Ta, at the strain rate of 0.0053 s^−1^, the yield stress decreases from 135 to 105 MPa when the temperature increases from 422 to 589 K; at 298 K, the yield stress increases from 110 to 280 MPa when the strain rate increases from 10^–6^ to 10^–1^ s^−1^. Then, for Ti–6Al–4V alloy, the working-hardening decreases when the temperature increases from 223 to 422 K at strain rate of 10^–3^ s^−1^; at 298 K, it also decreases when the strain rate increases from 10^–5^ to 1900 s^−1^.

The comparisons between calculated and experimental results also reflect that the present model describes the deformation behavior of different metal materials over wide ranges of temperatures and strain rates well. For metallic materials at higher strain rate, the fluctuation would appear around the yield point in the stress–strain curve. In the experiment, the amplitude of the fluctuation could change with test method, type of machine, testing environment and so on. The raising or lowering of stress at higher strain rate is not an inherent deformation behavior for materials. For a macroscopical constitutive model, the calculated stress–strain curve is usual smooth and continuous. Hence, the fluctuation of stress for BCC Ta at strain rate of 10^–1^ s^−1^ and 10^–2^ s^−1^ was not reflected by the present model.

## Discussion

The present model was established based on the new decomposition of deformation gradient. Firstly, the free thermal expansion of lattice was considered in the total deformation process and the thermal strain was introduced into the total strain. The thermal strain can be calculated easily based on the Eqs. (5) and (6). Then, the thermo-elastic constitutive equations for single crystals and polycrystalline metals were established. The elastic stiffness at different temperatures would be obtained by Eqs. (9) and (11). Hence, the present model, which couples the thermal vibration and the structural deformation together, is more concise and efficient than the atomistic models^20^ since that there is no need to compute the normal vibration frequency and the thermal effect is reflected by the thermal expansion of lattice.

For the plastic deformation behavior, the new relation between the stress and plastic strain is expressed by Eq. (14) with nine parameters. Although the number of parameters in the present model is a little more than JC and KHL models, the computational process of the present model is as simple as these two models. And the material parameters could be obtained easily by Matlab based on the stress–strain curves of experiments. More importantly, Eq. (14) can describe the strain rate and temperature effect on the plastic behavior of crystalline metallic materials accurately. Firstly, the strain-hardening index* m* is less than 1 and keeps the same when the strain rate and temperature change, which reveals that the work-hardening rate decreases with strain in the deformation process. Then, the parameter *γ *is less than zero, which reveals that the yield stress and working-hardening decrease with temperature. Lastly, since the parameters *A*, *B*, *C* and *n* are greater than or equal to 0, the *P*_*1*_, *P*_*2*_ and *β* would reflect the increase or decrease with strain rate in yield stress and working-hardening, which makes the present model able to describe the deformation behavior of crystalline metallic materials with various structure.

Therefore, the present model is better than JC and ZA models because the JC model is not appropriate for the material that the work-hardening decreases with increasing strain rate^13^ and the ZA model considers two different forms for fcc and bcc materials^38^. Comparing with KHL model, the advantage of the present model is the coupling of structural deformation and thermal vibration by the new decomposition of deformation gradient.

In the future work, considering that the deformation behavior of crystalline metallic materials is closely related to the microstructure evolution, the strain rate and temperature effects on the microscopic deformation mechanisms will be investigated combining with experimental results. Based on the present work, the change law of dislocation density with test temperature and strain rate will be analyzed by the microscopic observation results, and then it will be introduced into the relationship between the stress and plastic strain. A new constitutive model based on the microscopic deformation mechanisms will be established to not only describe the deformation behavior but also reveal the microscopic deformation mechanism for metallic materials. And the relationship between the macroscopical deformation behavior and microscopic mechanism will be revealed furtherly.

## Conclusion

The strain rate dependent thermo-elasto-plastic constitutive model in the present work was proposed to describe the effects of temperature and strain rate on the deformation behavior for crystalline metallic materials. The effect of free thermal expansion is introduced into this model by the new decomposition of the deformation gradient. The thermo-elastic constitutive equations of single crystal and polycrystal were established firstly, and then a new relation between the stress and plastic strain was proposed to describe the temperature and strain rate effects on the yield stress, as well as the work-hardening behavior of metallic materials. The present constitutive model gave an excellent correlation with the experimental data of different crystalline metallic materials over wide ranges of strain rates and temperatures. And it has good universality and high efficiency for crystalline metal materials.
